# The utilization and prognostic impact of B-type Natriuretic Peptide in hospitalized acute decompensated heart failure in an Asian population

**DOI:** 10.1186/s12872-016-0342-z

**Published:** 2016-09-09

**Authors:** Li Juen Chen, Chung-Lieh Hung, Hung-I Yeh, Mei-Jy Jeng, Cheng-Huang Su, Te-Yu Wu, Shou-Chuan Shih, Cheng-Ho Tsai

**Affiliations:** 1 Institute of Emergency and Critical Care Medicine, School of Medicine, National Yang-Ming University, No.155, Sec.2, Linong Street, Taipei, 112 Taiwan; 2 UW Medicine Valley Medical Center, 400 S 43rd Street, Renton, WA 98055 USA; 3 Department of Medicine, Mackay Medical College, No.46, Sec. 3, Zhongzheng Rd., Sanzhi Dist., New Taipei City, 252 Taiwan; 4 Division of Cardiology, Department of Internal Medicine, Mackay Memorial Hospital, Taipei Branch, No. 92, Sec. 2, Zhongshan N. Rd., Taipei City, 10449 Taiwan; 5 Department of Pediatrics, School of Medicine, National Yang-Ming University, No.155, Sec.2, Linong Street, Taipei, 112 Taiwan; 6 Department of Pediatrics, Taipei Veterans General Hospital, No. 201, Section 2, Shipai Rd, Beitou District, Taipei City, 112 Taiwan; 7 Division of Gastroenterology, Department of Internal Medicine, Mackay Memorial Hospital, Taipei Branch, No. 92, Sec. 2, Zhongshan N. Rd, Taipei City, 10449 Taiwan

**Keywords:** B-type natriuretic peptide, Acute decompensated heart failure, In-hospital mortality

## Abstract

**Background:**

B-type natriuretic peptide (BNP) levels during admission have been shown to have prognostic value in the diagnosis of heart failure and further predict the in-hospital mortality of acute decompensated heart failure (ADHF). This study describes the characteristics of BNP among hospitalized ADHF and elucidates its prognostic value of in-hospital mortality in an Asian population.

**Methods:**

We consecutively studied patients aged 20+ who were discharged with a diagnosis of ADHF from March 2013 to March 2014 in a tertiary hospital of northern Taiwan by reviewing medical records. Prognostic predictors of mortality were assessed using Cox proportional hazard regression models. BNP > 100 pg/ml was used as the cut-off for defining abnormally high BNP based on current clinical practice criteria.

**Results:**

After implementation of our exclusion criteria, a total of 1,807 patients hospitalized with ADHF were studied. Compared to those subjects with BNP ≤100 pg/ml, individuals with higher BNP tended to have more advanced age, more clusters of the typical signs of heart failure (HF) (e.g., peripheral edema or lung rales) at presentation, lower ejection fraction, lower hemoglobin levels, more disturbed biochemical data, worsened renal function, and twice the risk for in-hospital mortality (15.2 vs 6.2 %, all *p* < 0.05). In a multivariate analysis, more advanced age, the presence of rales, a worse New York Heart Association functional class, wider QRS duration, and abnormal BNP levels (>100 pg/ml) were all associated with in-hospital mortality among admitted HF patients after accounting for clinical co-variates and global ventricular ejection fraction (HR: 2.17, 95 % CI: 1.15–6.64, *p* = 0.024).

**Conclusion:**

Abnormally high BNP levels in ADHF patients during admission were tightly linked to clinical features of worse physical, functional, and clinical presentations, and further provided prognostic value for determining in-hospital mortality among patients with ADHF in an Asian population.

## Background

Multiple epidemiologic studies have demonstrated that advanced age [1], pulmonary basilar rales [2], dyspnea [3], QRS duration [4] cardiomegaly [5], higher urea [6], higher potassium [7, 8] lower sodium [9], higher B-type Natriuretic Peptide (BNP) [10], and lower hemoglobin [11] at presentation are all associated with in-hospital mortality among patients with acute decompensated heart failure (ADHF). BNP is among the most powerful prognostic markers in all forms of clinical heart failure (HF) [12]; it is recognized as a gold standard marker for outcome prediction in HF [11, 13–15]. Clinically relevant characteristics of Asian populations and the clinical prognostic factors with hospitalized ADHF have not been fully studied in a large observational cohort. The aims of this study are to explore the clinical characteristics relevant to abnormal BNP among unplanned hospitalized with ADHF and to further evaluate determinants of in-hospital mortality among ADHF in an Asian population.

## Methods

This retrospective study of ADHF used electronic medical records to investigate 1,807 consecutive patients aged 20+ who were hospitalized and discharged with a main clinical diagnosis of HF from March 2013 to March 2014 in an urban tertiary teaching hospital in Taiwan. Datasets were collected as routine medical management and hospitalization claims covered under the National Health Insurance Bureau of Taiwan. Heart failure was defined by using the European Society of Cardiology guidelines [16]. Hospitalization for heart failure was defined as unplanned presentation at our emergency department of patients receiving intravenous diuretic (e.g. loop diuretics, such as furosemide) or vasodilator treatment of new or worsening HF requiring admission to our hospital [17, 18]. Definition of ADHF in our current work was made by new onset or rapidly worsening HF signs and symptoms requiring urgent and unplanned hospitalization [15, 19, 20]. As in previous hospitalized acute HF studies, BNP >100 pg/ml was used as the cut-off for abnormally high BNP levels [21, 22]. Initial admission findings were mainly collected in the emergency department. Clinical information including cardiomegaly and data about left ventricular ejection fraction (LVEF) were collected from echocardiography. Pharmaceutical intravenous drugs administered at the emergency department were abstracted. Medical histories of chronic heart failure (CHF), hypertension, diabetes, coronary artery disease (CAD), chronic kidney disease (CKD), dyslipidemia, stroke, and anemia were abstracted as documented in electronic medical records. The main exclusion criteria were end stage renal disease or initial admission creatinine >3 mg/dl, acute coronary syndrome (including myocardial infarction or unstable angina), dialysis, terminal stage malignancy, liver cirrhosis, or life expectancy less than 6 months.

### Statistical analysis

Baseline characteristics of ADHF patients were expressed as mean (standard deviation) for continuous variables and frequency (percentage) for categorical variables. Student T tests were used to test the differences between groups for demographic characteristics and echocardiography-derived measures or indices. Chi-square or the Fisher exact test was used for comparisons of proportional distributions of cardiovascular risk factors between groups, as appropriate. The risk factors of all causes of in-hospital mortality were determined using univariate and multivariate Cox proportional hazard regression models. The cumulative probability of in-hospital mortality by the BNP groups (>100 or ≤ 100 pg/ml) was graphically presented using the Kaplan Meier survival function with comparison of cumulative events by the log-rank test. The relationship between in-hospital mortality and levels of BNP at admission was presented using locally weighted smoothing regression scatterplots. The trend of in-hospital mortality by quartiles (Q) of BNP were Q1 < 260.5, Q2 260.5 to 617, Q3 618 to 1340, Q4 1341+ pg/ml was graphically presented using the Kaplan Meier survival function with comparison of cumulative events by chi-square. A two-sided *p* value < 0.05 was considered as a statistically significant difference. All clinical variables *p* < 0.10 in univariate Cox model were entered into multivariate analysis. All statistical analyses were performed with Stata statistical software package (StataCorp. 2011. Stata Statistical Software: Release 12. College Station, TX: StataCorp LP.)

## Results

### Baseline demographic differences based on BNP level in admitted ADHF individuals

A total of 1807 patients participated in our cohort study: 927 were female (51.3 %), the mean age was 75.27 ± 14.12 years with ages ranging from 20+ to 103, and average BNP levels were 1028.11 ± 1136.22 pg/ml. The baseline characteristics stratified by BNP group are presented in Table 1. More advanced age (76.3 vs 72.3 years, *p* = 0.003) was observed at admission in patients with abnormally high levels of BNP (BNP > 100 pg/ml); no gender distribution differences was observed (*p* = 0.63). Initial admission findings show that pulmonary basilar rales, worse NYHA FC, peripheral edema, lower LVEF, higher urine acid, higher urea, higher creatinine, lower glomerular filtration rate (GFR), higher potassium, lower hemoglobin, and a higher prevalence of medical histories of CKD and A-fib were observed in those patients with BNP >100 pg/ml versus patients with BNP ≤100 pg/ml. Length of hospital stay and admission vital signs, including blood pressure and heart rates, showed no differences between the BNP ≤ 100 and >100 pg/ml groups. Univariate and multivariate analyses for clinical determinants of in-hospital mortality are presented in Table 2.

**Table 1:** Baseline characteristics categorized by BNP group

Variable	All patients (*n* = 1807)	BNP ≤ 100 (*n* = 130)		BNP > 100 (*n* = 1677)	*p*-value
Age (year)	75.27 (14.12)	72.29 (14.53)		76.25 (14.07)	0.003
Gender					
Female	927 (51.30 %)	65 (50.0 %)		876 (52.23 %)	0.630
Male	880 (48.69 %)	65 (50.0 %)		801 (47.77 %)	
Mortality					
Alive	1569 (86.83 %)	122 (93.85 %)		1421 (84.76 %)	0.005
Death	238 (13.17 %)	8 (6.15 %)		256 (15.24 %)	
Stay in hospital (day)	12.05 (40.71)	14.34 (20.79)		14.33 (35.6)	0.985
Initial admission findings					
Heart rate (beats/min)	90.27 (21.60)	91.83 (18.77)		92.81 (22.91)	0.584
SBP (mmHg)	135.87 (28.11)	135.59 (28.45)		136.33 (29.46)	0.786
DBP (mmHg)	74.26 (17.53)	73.26 (17.09)		74.41 (18.58)	0.501
Rales	599 (33.15 %)	36 (27.69 %)		467 (41.62 %)	0.002
PND	39 (2.15 %)	2 (1.54 %)		35 (3.12 %)	0.314
Orthopnea	138 (7.64 %)	7 (5.38 %)		122 (10.87 %)	0.051
NYHA FC (III-IV)	591 (32.70 %)	45 (34.62 %)		475 (42.34 %)	0.091
Peripheral edema	473 (26.18 %)	30 (23.08 %)		357 (31.82 %)	0.041
QRS duration (ms)	101.91 (24.92)	99.73 (23.01)		102.94 (25.44)	0.141
LVEF (%)	54.33 (13.70)	60.05 (9.68)		52.97 (14.09)	<0.001
Cardiomegaly	667 (36.91 %)	56	(43.08 %)	516 (45.99 %)	0.528
Glucose (mg/dl)	168.35 (93.67)	172.16 (101.59)		178.56 (96.64)	0.480
HbA1c (%)	6.75 (1.74)	6.64 (1.68)		6.80 (1.75)	0.649
Total bilirubin (mg/dl)	1.31 (2.21)	0.93 (0.59)		1.38 (2.49)	0.072
GPT (u/l)	37.50 (70.82)	35.98 (63.76)		41.52 (80.69)	0.268
Urine acid (mg/dl)	7.32 (2.79)	6.62 (2.25)		7.72 (2.83)	<0.001
Urea (mg/dl)	28.63 (17.92)	24.59 (17.02)		30.42 (18.00)	<0.001
Creatinine (mg/dl)	1.46 (0.74)	1.28 (0.65)		1.54 (0.76)	<0.001
GFR (ml/min/1.73 m^2^)	54.0 (30.90)	62.38 (31.14)		50.27 (28.89)	<0.001
Potassium (meq/l)	4.08 (0.77)	3.89 (0.64)		4.09 (0.81)	0.001
Sodium (meq/l)	136.62 (6.14)	136.67 (5.65)		136.31 (6.42)	0.547
Troponin (ng/ml)	0.51 (3.62)	0.22 (1.68)		0.57 (4.06)	0.340
BNP (pg/ml)	1028.11 (1136.22)	46.81 (27.13)		1141.81 (1147.18)	<0.001
Hemoglobin (g/dl)	11.40 (2.57)	12.29 (2.70)		11.25 (2.54)	<0.001
Medical history (*n*, %)					
CHF	1050 (58.11 %)	62 (47.69 %)		988 (54.63 %)	0.133
Hypertension	1100 (60.87 %)	81 (62.31 %)		1019 (59.36 %)	0.516
Diabetes	752 (41.62 %)	59 (45.38 %)		693 (42.25 %)	0.493
CAD	435 (24.07 %)	27 (20.77 %)		408 (22.37 %)	0.677
CKD	395 (21.86 %)	18 (13.85 %)		377 (23.89 %)	0.010
Dyslipidemia	376 (20.81 %)	30 (23.08 %)		346 (18.72 %)	0.232
Stroke	300 (16.60 %)	30 (23.08 %)		270 (17.91 %)	0.151
A-fib	396 (21.91 %)	13 (10.0 %)		383 (24.24 %)	<0.001
Anemia	167 (9.24 %)	9 (6.92 %)		158 (9.98 %)	0.264
Hepatitis	38 (2.10 %)	4 (3.08 %)		34 (2.23 %)	0.543
Medications (*n*, %)					
Diuretics	1432 (79.26 %)	85 (65.38 %)		1347 (80.32 %)	<0.001
Nitrates	844 (46.7 %)	91 (70.00 %)		753 (44.90 %)	<0.001
Digoxin	87 (4.86 %)	4 (3.08 %)		83 (4.95 %)	0.337
Beta blocker	72 (3.98 %)	0 (0 %)		72 (4.29 %)	0.016

*Abbreviations*: *SBP* systolic blood pressure, *DBP* diastolic blood pressure, *PND* paroxysmal nocturnal dyspnea, *NYHA FC* New York Heart Association functional class, *LVEF* left ventricular ejection fraction, *HbA1c* glycated hemoglobin, *GPT* glutamate pyruvate transaminase, *GFR* glomerular filtration rate, *BNP* B-type natriuretic peptide, *CHF* chronic heart failure, *CAD* coronary artery disease, *CKD* chronic kidney disease, A-fib atrial fibrillation

**Table 2:** Predictors of in-hospital mortality (univariate and multivariate analysis)

	Univariate			Multivariate		
Variables	HR	[95 % C.I. for HR]	*P*-value	HR	[95 % C.I. for HR]	*P*-value
Age (year)	1.692	(1.152, 2.486)	0.007	1.020	(1.01, 1.03)	0.007
Gender (Male/Female)	1.102	(0.849, 1.430)	0.464			
Heart rate (beats/min)	1.004	(0.999, 1.009)	0.152			
SBP (mmHg)	0.997	(0.993, 1.001)	0.177			
DBP (mmHg)	0.995	(0.988, 1.003)	0.223			
Rales	1.709	(1.317, 2.218)	<0.001	1.500	(1.065, 2.114)	0.02
PND	0.526	(0.131, 2.121)	0.367			
Orthopnea	0.645	(0.359, 1.160)	0.143			
NYHA FC (III-IV)	1.980	(1.436, 2.433)	<0.001	1.662	(1.288, 3.052)	0.002
Peripheral edema	0.824	(0.595, 1.141)	0.243			
QRS duration (ms)	1.007	(1.002, 1.012)	0.007	1.010	(1.001, 1.01)	0.023
LVEF (%)	1.002	(0.988, 1.015)	0.812			
Cardiomegaly	1.505	(1.151, 1.969)	0.003	0.945	(0.617, 1.448)	0.796
Glucose (mg/dl)	0.999	(0.998, 1.001)	0.359			
HbA1c (%)	0.801	(0.627, 1.023)	0.076			
Total bilirubin (mg/dl)	1.052	(1.009, 1.098)	0.018	1.037	(0.978, 1.099)	0.222
GPT (u/l)	1.000	(0.998, 1.001)	0.618			
Urine acid (mg/dl)	1.046	(0.994, 1.101)	0.081			
Creatinine (mg/dl)	1.106	(0.939, 1.304)	0.227			
GFR (ml/min/1.73 m^2^)	1.000	(0.996, 1.005)	0.833			
Potassium (meq/l)	1.262	(1.109, 1.436)	<0.001	1.125	(0.939, 1.347)	0.202
Sodium (meq/l)	0.978	(0.961, 0.995)	0.010	1.000	(0.973, 1.027)	0.979
Troponin (ng/ml)	1.004	(0.963, 1.046)	0.864			
BNP (>100/≤100)	2.486	(1.218, 5.071)	0.012	2.170	(1.145, 6.640)	0.024
Hemoglobin (g/dl)	0.904	(0.856, 0.954)	<0.001	0.971	(0.901, 1.046)	0.433
CHF	0.779	(0.600, 1.012)	0.161			
Hypertension	0.672	(0.517, 0.872)	0.303			
Diabetes	0.770	(0.587, 1.009)	0.158			
CAD	0.801	(0.558, 1.149)	0.228			
CKD	0.565	(0.380, 0.839)	0.505			
Dyslipidemia	1.321	(0.967, 1.806)	0.180			
Stroke	0.942	(0.673, 1.317)	0.726			
A-fib	0.455	(0.306, 0.676)	0.101			
Anemia	0.655	(0.394, 1.089)	0.103			
Hepatitis	1.351	(0.666, 2.739)	0.404			

*Abbreviations*: *SBP* systolic blood pressure, *DBP* diastolic blood pressure, *PND* paroxysmal nocturnal dyspnea, *NYHA FC* New York Heart Association functional class, *LVEF* left ventricular ejection fraction, *HbA1c* glycated hemoglobin, *GPT* glutamate pyruvate transaminase, *GFR* glomerular filtration rate, *BNP* B-type natriuretic peptide, *CHF* chronic heart failure, *CAD* coronary artery disease, *CKD* chronic kidney disease, A-fib atrial fibrillation

### Clinical predictors for in-hospital mortality

**Fig. 1:**
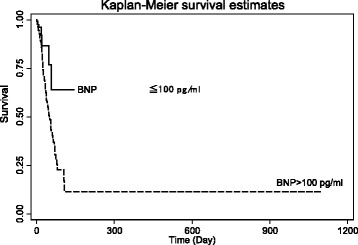
Kaplan-Meier survival curves by BNP group. (The log-rank test: *χ*2 = 6.78; *p*-value = 0.0092)

**Fig. 2:**
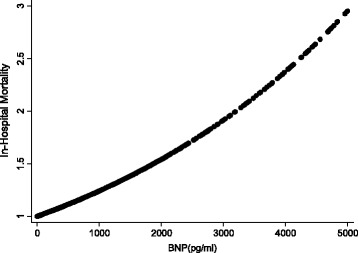
Scatterplot Smoother of admission BNP levels and in-hospital mortality

**Fig. 3:**
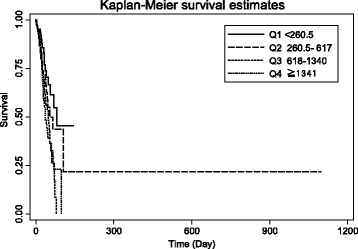
Kaplan-Meier survival curves for BNP quartiles. Test for trend of survival functions: Chi-square = 11.14; *P* = 0.0008

In a univariate Cox model, advanced age, presentation at admission with pulmonary basilar rales, worse NYHA FC, cardiomegaly, wider QRSD, higher total bilirubin, higher potassium, lower sodium, abnormally high BNP and lower hemoglobin were all associated with in-hospital mortality among ADHF patients (all *p* < 0.05). Multivariate Cox proportional hazard regression analysis was further performed to determine the independent risk factors associated with in-hospital mortality. After adjusting for age, gender, rales, NYHA FC, QRSD, cardiomegaly, total bilirubin, urine acid, urea, potassium, sodium, BNP, and hemoglobin we found that more advanced age (HR: 1.02, 95 % CI: 1.01–1.03), presence of rales (HR: 1.50, 95 % CI: 1.07–2.11), worse NYHA FC (HR: 1.66, 95 % CI: 1.29–3.05), more widened QRS duration (HR: 1.01, 95 % CI: 1.001–1.01) and admission with abnormally high BNP (>100 pg/ml, HR: 2.17, 95 % CI: 1.15–6.64) were associated with in-hospital mortality among hospitalized ADHF (all *p* < 0.05). Our patients in the higher BNP group (BNP >100 pg/ml) had a graver prognosis than their lower (BNP ≤100 pg/ml) counterparts. Kaplan-Meier survival curves by BNP group are presented in Fig. 1. (The log-rank test: *χ*2 = 6.78; *p*-value = 0.0092). A local regression scatterplot smoothing of BNP levels and in-hospital mortality risk is presented in Fig. 2—further confirming BNP as a continuous clinical biomarker and a predictor of in-hospital mortality. Kaplan-Meier survival curves by quartiles (Q) of BNP were Q1 < 260.5 pg/ml, Q2 (260.5–617 pg/ml), Q3 (618–1340 pg/ml), Q4 ≥ 1341 are presented in Fig. 3. Mortality rates in the BNP quartiles were tested for trend of survival functions: Chi-square = 11.23; *p*-value = 0.0008. Higher BNP quartiles predicted higher in-hospital mortality.

## Discussion

Our cohort study revealed several findings: abnormally high BNP levels, advanced age, pulmonary basilar rales, worse NYHA FC, and a wider QRS duration were independently associated with in-hospital mortality among hospitalized ADHF patients. Admission BNP levels were independently associated with in-hospital mortality. Krim et al. [20] reported that in a large cohort in North America with 92,072 participants from 264 hospitals, BNP levels at admission in Asian patients averaged 1066 pg/ml; for blacks 866 pg/ml; for whites 776 pg/ml; and for Hispanics 737 pg/ml. BNP levels at admission were 1028 pg/ml in our study, which was comparable to previous studies. Previous data have proposed that BNP levels may vary with genetic factors and that the higher BNP levels observed in Asian populations may be related to higher rates of renal insufficiency [20]. Indeed, Taiwan has the highest incidence and prevalence rates of end stage renal disease and a high prevalence of CKD [23], making renal insufficiency a likely cause of the comparatively high average BNP levels in our cohort.

From the large observational database ADHERE (Acute Decompensated Heart Failure National Registry)—a registry contained data on patients hospitalized with acute decompensated HF from 191 hospitals across United States of America—Fonarow et al. [22] reported that BNP levels at admission independently predicted mortality in ADHF patients hospitalized with reduced and preserved systolic function. To the best of our knowledge, data regarding the clinical presentation and in-hospital prognostic information using prior BNP in an admitted HF cohort remained largely unknown in large Asian populations. Our analysis of a relatively large group of HF hospitalizations confirmed that admission BNP levels can be a reliable and accountable predictor of the risk of in-hospital mortality among hospitalized ADHF. Previously, many other publications reported that BNP contributed independently to the risk prediction of in-hospital mortality. Our analysis shows that admission levels of BNP give prognostic information about patients hospitalized for ADHF. Moreover, it is the first to report in an Asian population. BNP has been globally used to assist clinical decision-making and to critically appraise patients with HF. However, interpretation of BNP measurements also depends on many factors including obesity, CKD, the status of congestion or decongestion, specific treatments, and race/ethnicity among hospitalized ADHF patients [12]. Similar to prior findings, our work showed that several key clinical co-variates, including worse NYHA FC, presence of rales, wider QRS duration, as well as abnormally high BNP (set at 100 pg/ml) remained as independent prognosticators of in-hospital mortality.

Careful clinical evaluation/reasoning and interpretation of laboratory results in the correct context will always add to laboratory tests alone in patients with complex syndrome of HF. Clinical decision-making involves many factors, including understanding scientific evidence, experience, and the patient’s profile. Even if levels of BNP are easily available, yield quick results, and are widely used, they still do not contribute explicit guidance regarding specific HF therapies. Rapid and early patient identification may aid faster progressive therapies that improve a patient’s symptoms and enable an estimate of the combined end point of death and failure to achieve a favorable health status [24]. Our analyses in this observational cohort suggest rales and worse NYHA FC (two congestive signs on physical assessment) were independently associated with in-hospital mortality. Worse functional class has been the most common sign and symptom of heart failure causing patients to seek medical treatment at emergency departments. Early dyspnea relief had been demonstrated to be associated in reduced mortality Dyspnea is an important marker of congestion and a target for treatment. However, there is no validated method of measuring dyspnea in HF patients [25]. A post-hoc analysis of the AF-CHF (Atrial Fibrillation and Chronic Heart Failure) found that rales was associated with mortality among other congestive signs including peripheral edema, jugular venous distension, and a third sound [2]. Our study and the AF-CHF Trial continue to show that congestive signs on physical assessment have important prognostic value in HF patients. A retrospective analysis of the CIBIS-II (Cardiac Insufficiency Bisoprolol Study – II) in 2647 patients with NYHA functional class III to IV symptoms, found that congestive signs of jugular venous distension and peripheral edema were associated with HF in-hospital mortality [26]. Importantly, rale as a congestive sign on physical assessment was not evaluated [2]. Further, wide QRS duration had also recently been proved to be a key prognosticator in HF survival and hospitalization [27]. In line with these prior reports, our study demonstrated that admission levels of BNP, advanced age, worse NYHA FC, wider QRS duration and pulmonary rales were the strongest risk factors for mortality among unplanned hospitalized with ADHF. These analyses suggest that conventional physical assessments in this modern era continue to provide important prognostic value over the more routine clinical parameters of electrocardiography and echocardiography, especially LVEF [2].

There were a total of 7 % admitted HF subjects who presented with BNP levels < 100 pg/ml in our current study. Fonarow et al. reported a 3.3 % prevalence of HF subjects with BNP levels <100 pg/ml from ADHERE (Acute Decompensated Heart Failure National Registry) from 48,629 hospitalizations [22]. A previous study reported that BNP levels provided the accurate diagnosis of 81.1 % (sensitivity of 90 % and specificity of 74 %) at a cutoff of >100 pg/ml among congestive heart failure patients presenting at the emergency department [21]. In our study and a report from ADHERE [22], patients in the higher BNP quartiles had a higher trend for mortality. Thus admission levels of BNP allow the acquisition of expedited prognostic information regarding in-hospital mortality for hospitalized ADHF patients. Based on our findings, rapid and early recognition of several specific risk factors may benefit a subgroup of patients for whom aggressive optimization of drug or device therapies could improve their outcomes and further provide possible beneficial interventions to improve their in-hospital survival during admission.

### Limitations

The results of this observational study should be interpreted in the context of several limitations. This is a single center study with retrospective manner and may not represent the general population. Although consecutiveness of enrollment was rigidly recommended, a hospitalized patient census was not checked or available. Therefore, we cannot prove the consecutiveness of patient enrollment. The accuracy and completeness of collected dataset depends on the original documenters. Patients were mainly recruited from the cardiology and pulmonary departments, and thus might not represent the general population. We did not report oral medications at admission. The dataset of home regimen medications was not complete or unavailable.

## Conclusion

Admission BNP levels remained a prognostic value for determining in-hospital mortality among patients with unplanned hospitalization with ADHF in our Asian population. The prognostic value of BNP in such a patient population was also shown to be independent of other clinical or lab data, including echocardiography. Routine clinical implementation of biomarkers for any admitted HF patient has therefore been proposed to better understand the pathophysiological mechanisms and to offer not only a more accurate prediction of the specific patient subgroup at higher risk for unfavorable in-hospital events but also facilitate the introduction of effective therapy, if needed, in a timely fashion.

## Key messages

1. Admission BNP levels provided prognostic value for determining in-hospital mortality among patients with unplanned hospitalization with ADHF in our Asian population.

2. Congestive signs of pulmonary rales and dyspnea on the physical assessment continue to provide prognostic value.

## Abbreviations

ADHF: Acute decompensated heart failure
BNP: B-type natriuretic peptide
CAD: Coronary artery disease
CHF: Chronic heart failure
CKD: Chronic kidney disease
DBP: Diastolic blood pressure
GFR: Glomerular filtration rate
GPT: Glutamate pyruvate transaminase
HbA1c: Glycated hemoglobin
LVEF: Left ventricular ejection fraction
NYHA FC: New York Heart Association functional class
PND: Paroxysmal nocturnal dyspnea
SBP: Systolic blood pressure

## References

[1] Mosterd A, Hoes AW (2007). Clinical epidemiology of heart failure. Heart.

[2] Caldentey G, Khairy P, Roy D, Leduc H, Talajic M, Racine N (2014). Prognostic value of the physical examination in patients with heart failure and atrial fibrillation: insights from the AF-CHF trial (atrial fibrillation and chronic heart failure). JACC Heart Fail.

[3] Metra M, O’Connor CM, Davison BA, Cleland JG, Ponikowski P, Teerlink JR (2011). Early dyspnoea relief in acute heart failure: prevalence, association with mortality, and effect of rolofylline in the PROTECT Study. Eur Heart J.

[4] Wang NC, Maggioni AP, Konstam MA, Zannad F, Krasa HB, Burnett JC (2008). Clinical implications of QRS duration in patients hospitalized with worsening heart failure and reduced left ventricular ejection fraction. JAMA.

[5] Kearney MT, Fox KA, Lee AJ, Prescott RJ, Shah AM, Batin PD (2002). Predicting death due to progressive heart failure in patients with mild-to-moderate chronic heart failure. J Am Coll Cardiol.

[6] Yancy CW, Lopatin M, Stevenson LW, De Marco T, Fonarow GC, ADHERE Scientific Advisory Committee and Investigators (2006). Clinical presentation, management, and in-hospital outcomes of patients admitted with acute decompensated heart failure with preserved systolic function: a report from the Acute Decompensated Heart Failure National Registry (ADHERE) Database. J Am Coll Cardiol.

[7] Jain N, Kotla S, Little BB, Weideman RA, Brilakis ES, Reilly RF (2012). Predictors of hyperkalemia and death in patients with cardiac and renal disease. Am J Cardiol.

[8] Spinar J, Parenica J, Vitovec J, Widimsky P, Linhart A, Fedorco M (2011). Baseline characteristics and hospital mortality in the Acute Heart Failure Database (AHEAD) Main registry. Crit Care.

[9] Rusinaru D, Tribouilloy C, Berry C, Richards AM, Whalley GA, Earle N (2012). Relationship of serum sodium concentration to mortality in a wide spectrum of heart failure patients with preserved and with reduced ejection fraction: an individual patient data meta-analysis (†): Meta-Analysis Global Group in Chronic heart failure (MAGGIC). Eur J Heart Fail.

[10] Logeart D, Thabut G, Jourdain P, Chavelas C, Beyne P, Beauvais F (2004). Predischarge B-type natriuretic peptide assay for identifying patients at high risk of re-admission after decompensated heart failure. J Am Coll Cardiol.

[11] Lourenço P, Ribeiro A, Pintalhão M, Silva S, Bettencourt P (2015). Predictors of Six-month mortality in BNP-matched acute heart failure patients. Am J Cardiol.

[12] Francis GS, Felker GM, Tang WH (2016). A test in context: critical evaluation of natriuretic peptide testing in heart failure. J Am Coll Cardiol.

[13] Januzzi JL, van Kimmenade R, Lainchbury J, Bayes-Genis A, Ordonez-Llanos J, Santalo-Bel M (2006). NT-proBNP testing for diagnosis and short-term prognosis in acute destabilized heart failure: an international pooled analysis of 1256 patients: the International Collaborative of NT-proBNP Study. Eur Heart J.

[14] Latini R, Masson S, Wong M, Barlera S, Carretta E, Staszewsky L (2006). Incremental prognostic value of changes in B-type natriuretic peptide in heart failure. Am J Med..

[15] Maisel AS, Krishnaswamy P, Nowak RM, McCord J, Hollander JE, Duc P (2002). Rapid measurement of B-type natriuretic peptide in the emergency diagnosis of heart failure. N Engl J Med.

[16] Cao TH, Quinn PA, Sandhu JK, Voors AA, Lang CC, Parry HM (2015). Identification of novel biomarkers in plasma for prediction of treatment response in patients with heart failure. Lancet.

[17] Shin SH, Hung CL, Uno H, Hassanein AH, Verma A, Bourgoun M (2010). Mechanical dyssynchrony after myocardial infarction in patients with left ventricular dysfunction, heart failure, or both. Circulation.

[18] Senni M, Gavazzi A, Oliva F, Mortara A, Urso R, Pozzoli M (2014). In-hospital and 1-year outcomes of acute heart failure patients according to presentation (de novo vs. worsening) and ejection fraction. Results from IN-HF Outcome Registry. Int J Cardiol.

[19] Gheorghiade M, Pang PS (2009). Acute heart failure syndromes. J Am Coll Cardiol.

[20] Krim SR, Vivo RP, Krim NR, Qian F, Cox M, Ventura H (2013). Racial/Ethnic differences in B-type natriuretic peptide levels and their association with care and outcomes among patients hospitalized with heart failure: findings from Get With The Guidelines-Heart Failure. JACC Heart Fail.

[21] Morrison LK, Harrison A, Krishnaswamy P, Kazanegra R, Clopton P, Maisel A (2002). Utility of a rapid B-natriuretic peptide assay in differentiating congestive heart failure from lung disease in patients presenting with dyspnea. J Am Coll Cardiol.

[22] Fonarow GC, Peacock WF, Phillips CO, Givertz MM, Lopatin M, ADHERE Scientific Advisory Committee and Investigators (2007). Admission B-type natriuretic peptide levels and in-hospital mortality in acute decompensated heart failure. J Am Coll Cardiol.

[23] Hwang SJ, Tsai JC, Chen HC (2010). Epidemiology, impact and preventive care of chronic kidney disease in Taiwan. Nephrology (Carlton).

[24] Allen LA, Gheorghiade M, Reid KJ, Dunlay SM, Chan PS, Hauptman PJ (2011). Identifying patients hospitalized with heart failure at risk for unfavorable future quality of life. Circ Cardiovasc Qual Outcomes.

[25] Gheorghiade M, Follath F, Ponikowski P, Barsuk JH, Blair JE, Cleland JG (2010). Assessing and grading congestion in acute heart failure: a scientific statement from the acute heart failure committee of the heart failure association of the European Society of Cardiology and endorsed by the European Society of Intensive Care Medicine. Eur J Heart Fail.

[26] Damman K, Voors AA, Hillege HL, Navis G, Lechat P, van Veldhuisen DJ (2010). Congestion in chronic systolic heart failure is related to renal dysfunction and increased mortality. Eur J Heart Fail.

[27] Cannon JA, Collier TJ, Shen L, Swedberg K, Krum H, Van Veldhuisen DJ (2015). Clinical outcomes according to QRS duration and morphology in the Eplerenone in Mild Patients: Hospitalization and SurvIval Study in Heart Failure (EMPHASIS-HF). Eur J Heart Fail.

